# An Augmented Reality Based Human-Robot Interaction Interface Using Kalman Filter Sensor Fusion

**DOI:** 10.3390/s19204586

**Published:** 2019-10-22

**Authors:** Chunxu Li, Ashraf Fahmy, Johann Sienz

**Affiliations:** 1 ASTUTE 2020 (Advanced Sustainable Manufacturing Technologies), Swansea University, Swansea SA1 8EN, UK; A.A.Fahmy@Swansea.ac.uk (A.F.); J.Sienz@Swansea.ac.uk (J.S.); 2 School of Automation and Electrical Engineering, Qingdao University of Science and Technology, Qingdao 266042, China; 3 Department of Electrical Power and Machines, Helwan University, Helwan 11795, Egypt

**Keywords:** augmented reality, human-robot interaction, LeapMotion sensor, Kinect sensor, Kalman filter sensor fusion

## Abstract

In this paper, the application of Augmented Reality (AR) for the control and adjustment of robots has been developed, with the aim of making interaction and adjustment of robots easier and more accurate from a remote location. A LeapMotion sensor based controller has been investigated to track the movement of the operator hands. The data from the controller allows gestures and the position of the hand palm’s central point to be detected and tracked. A Kinect V2 camera is able to measure the corresponding motion velocities in x, y, z directions after our investigated post-processing algorithm is fulfilled. Unreal Engine 4 is used to create an AR environment for the user to monitor the control process immersively. Kalman filtering (KF) algorithm is employed to fuse the position signals from the LeapMotion sensor with the velocity signals from the Kinect camera sensor, respectively. The fused/optimal data are sent to teleoperate a Baxter robot in real-time by User Datagram Protocol (UDP). Several experiments have been conducted to test the validation of the proposed method.

## 1. Intruduction

### 1.1. Background

Rapid development in robot technologies has necessitated the development of the robot’s locomotive capacities. In light of the myriad of uses, robots can serve in modernized hospitals, schools, business and other fields, the research regarding human-robot skill transfer has flourished during the last decades. Currently, robots are already being utilized with greater efficiency in the industrial field and it has been made possible to transfer human motor skills to robots after the motion signals of the operator have been captured by a computer. According to Reference [1], the human-robot interaction (HRI) is a sub-area of the human-computer interaction (HCI), which studies the interaction between humans and robots and focuses on developing more intelligent and anthropomorphic robots. HRI is widely used in the research and implementation of robot systems in hazardous zones where human involvement needs to be minimized and remote operation of robots is required. These inventions can also apply to care for the elderly and the disabled as well as for entertainment purposes [2]. There are various methods of HRI, among which, two are considered the main methods—physical interaction and teleoperation interaction.

Generally, the objective of HRI is to enable robots to have human motion capabilities; by doing this, robots can manipulate their movements with better dexterity and versatility like human beings [3]. To this end, two approaches have been adopted. One is by modelling human motor control which can be adapted and implemented on a robot. The other is to facilitate robot learning through demonstration, in which the human operator plays the role of a tutor and the robot, a tutee [4]. Development in this field started with the fixed trajectory based skills transfer from humans to robots. Thereafter, a hybrid mode was developed combining both position and force, which was further upgraded into an interactive mode by involving online feedback [5]. Recent progress includes the enhanced skill transfer via the use of bio-signals and physiological signals of the human body. These signals can provide us with a richer and deeper account of the mechanism for human muscle activities compared with physical signals [6]. Nowadays, with the advance of technology for HRI, demand for human operators has increased. Meanwhile, robot’s training processes have also significantly reduced. In Reference [7], an approach was developed for transferring skills between tutor and tutee by capturing the movements of the tutor on a sensory-based computational model. This information was then used to produce online master commands for developing the learning process of the less-skilled tutees. In other research [8], a research group employed a neural network to record human skills on a computer and then tried different ways to minimize the time taken for transferring skills from a human tutor to a tutee.

In recent years, motion capture technology has been developed and utilized in a wide range of areas, including HRI, computer animation and 3D film production. It serves to accurately record the 3D motion trajectory of each part of a moving object. Based on this information, accurate modelling of moving objects and concurrent semantic analyses of the movements can be achieved, which is of great help in technological fields such as animation [9]. In some special cases, for example the hazardous/toxic chemical manufacturing process or medical operations, robots are required to carry out tasks remotely and not under closed supervision by human operators for safety considerations. To this end, the traditional HRI methods and automation strategies are not applicable because the former requires a shared workspace between human operator and the robot, the latter is in a completely non-supervisory environment and lacks flexibility in terms of the emergent situation. Moreover, motion capture is an extremely time-consuming process, due to the fact that the captured movement data require a huge amount of both pre-processing and manual segmentation of the action sequences and also requires the accurate identification of semantics for each segment, which can cause the delaying problem. Sensor noises also exist in the tracking process.

**Figure 1:**
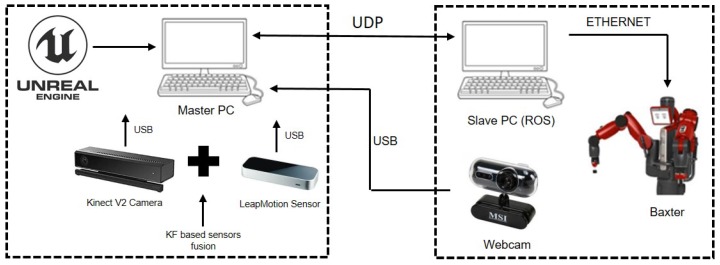
Flowchart of the proposed control system.

In this paper, our proposed control strategy employs the AR based teleoperation method to monitor and control the robot in real-time, the Kalman filtering (KF) method to fuse the signals from the LeapMotion sensor and a Kinect V2 sensor, in order to reduce the sensor noises and improve the tracking accuracy. Figure 1 shows the control block of our designed system.

### 1.2. Related Works

Mixed reality technology has attracted wide research interest in recent times. As a new computer technology, it has been applied in various fields. In Reference [10], authors developed an augmented reality-aided tele-presence system for robot manipulation for the usage of industrial manufacturing, where a robot was successfully controlled remotely to pick a known object from a starting position and placing it in different target positions. In Reference [11], a telepresence system that allows the immsersive remote control of a working machine was developed, where an omnidirectional stereo vision head mounted on the machine to interact with the operator who wears a head-mounted display. In Reference [12], the authors developed a novel wearable controller with haptic function which is suitable for various robotic platforms. According to Reference [13], authors developed an AR based interface for telerobotic operation using two robot programming modalities—free space trajectory and contact surface trajectory. In Reference [14], the authors investigated an AR operator interface for telerobotic operation based on Robot Operating System (ROS). All the above research has enhanced user experiences; however, they have not taken the sensor noises into account.

Since the data obtained from multiple sensors’ fusion are more accurate than a single sensor, in recent years, multi-sensor information fusion technology has been widely researched in many domains. In [15], authors developed a data fusion based multiple Kinect cameras framework that allows the human operator to interact with a robot safely. According to [16], human motion was tracked in real-time by multiple depth camera sensors and the KF sensor fusion was used to estimate the kinematic configuration of the human operator. In addition, particle filter algorithms are able to estimate the human pose [17], authors validated the proposed method on a realistic robotic platform. It can be concluded that, after the employment of the multiple sensors fusion technologies, the system becomes more robust. However, that research can also be promoted by adding the immersive monitoring technologies to enhance user experiences.

The above-mentioned research employed the KF algorithm to fuse homogeneous type data from multiple sensors such as the Kinect camera sensor. In the practical situation, the information output by Kinect is accompanied by noises, which can even be filtered out by data smoothing filtering, which results in a worse real-time performance of the system and these interferences are not able to obtain an accurate angular value. For multiple sensors fusion, according to Reference [18], the heterogeneous data fusion has stronger reliability than the homogeneous data fusion. Compared with the traditional filtering and robotic teleoperation methods, the contributions of our paper can be summarized as follows:

(1) the position from the LeapMotion was fused with the corresponding motion velocity instead of the position data measured by Kinect V2 sensor to improve the reliability of our proposed HRI interface;
(2) the AR telepresence was designed for the robot teleoperation to enhance user experiences.

## 2. System Description and Theory

### 2.1. Unreal Engine Base Augmented Reality Technology

According to Reference [19], augmented reality is a technology that combines virtual information with the real world. It uses a variety of techniques such as multimedia, 3D modeling, real-time tracking and registration, intelligent interaction and sensing. The computer-generated text, image, 3D model, music, video and other virtual information are simulated and applied to the real world. Google Glass in 2013, which is a wearable technology that used a small screen attached to a wearable structure to overlay useful information onto a person’s view and can be interacted with via human voice [20]. Compared with Virtual Reality, AR attracts more people because it is a reasonable complement to people’s daily lives. AR is a practical application of superimposed computer-generated virtual world in the real world. Various practical applications in medicine, education and industry have proved that AR has a significant impact on humans. It does not create a completely virtual world outside of the real world compared to VR.

Unreal Engine 4 is a complete suite of game development tools, see Reference [21]. It is mostly used for the creation of numerous video games but is also used in other applications such as training simulations. This development environment provides two different programming methods. Blueprint programming is a block based programming method that allows the user to use pre-made function blocks provided by Unreal to create a programme by linking blocks together, as well as adding other blocks for items such as variables [22]. The engine also supports raw C++ coding which gives more flexibility and completely customised functions; however, greater programming knowledge is required compared to when using the Blueprint coding. Unreal Engine supports plug-ins from other developers such as LEAP Motion, making it easy to interface external software and devices with Unreal. This also gives the user pre-made Blueprint function blocks for use with these devices pre-made by the developers.

### 2.2. Augmented Reality Environment Generation

In this paper, the Unreal Engine 4 Editor is used to create an AR environment, wherein the LeapMotion plug-in needs to be built into Unreal, which enables the functions of LeapMotion sensor in the Unreal Engine. Following this, the next step is to create a new *Blueprint* in *LeapGameMode*, which activates the LeapMotion characters as its default pawn. According to Reference [23], a pawn is an actor that can be controlled by either the player or the games AI. In our paper, the selected pawn is *LeapFloatingHandsCharacter*, which is a character easy to manipulate for floating hand models. Once the game mode has been successfully established, a webcam needs to be fed from the outside world into the AR environment,; to this end, *Unreal4AR* plug-in is employed. In addition, there is also a tool named *ARToolkitBase* which can be used for the view adjustment, such as the size of the display window and the position of the display window in the Unreal environment.

**Figure 2:**
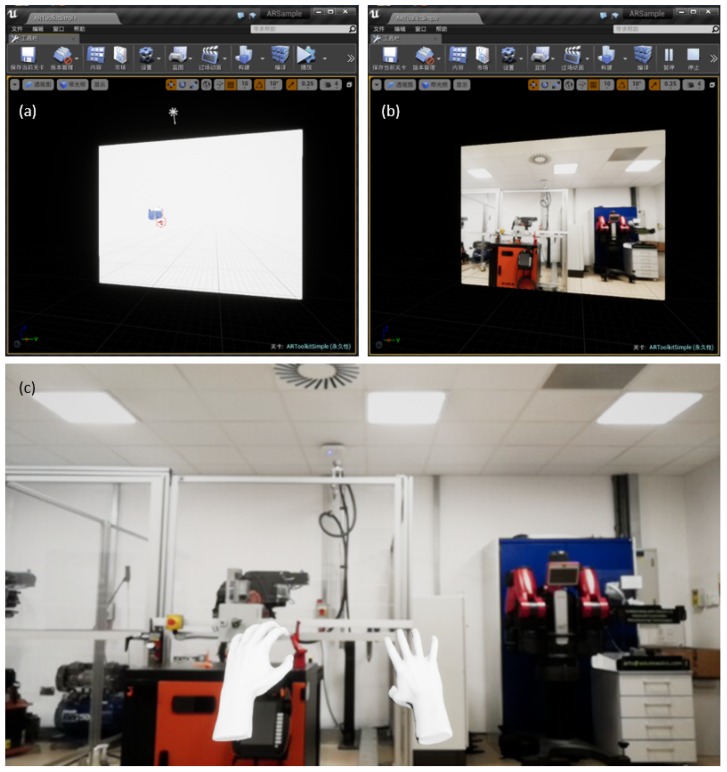
The Combination of LeapMotion Hands and the augmented reality (AR) environment: (**a**) Programme display without running; (**b**) Running programme display window without LeapMotion Hands; (**c**) Running programme display window with LeapMotion Hands in full screen size.

Up to now, a pair of virtual hands generated by the LeapMotion plug-in, and the display window fed with the webcam in the Unreal environment, have been obtained. The next stage is combining them. For this reason, a lesser LeapMotion hand component *LeapRiggedEchoHandsActor* is required, which lays the hands in the correct positions in front of the virtual camera component of the *Blueprint* [24]. By doing this, the hands are created as an item in the Unreal Engine game mode and are displayed persistently. So far, when we run the project created by Unreal Engine 4 Editor, the human operator could finally see their hands in the camera view (shown in Figure 2) with full LeapMotion movable functions.

### 2.3. Hand Tracking Using Leap Motion & Kinect

**Figure 3:**
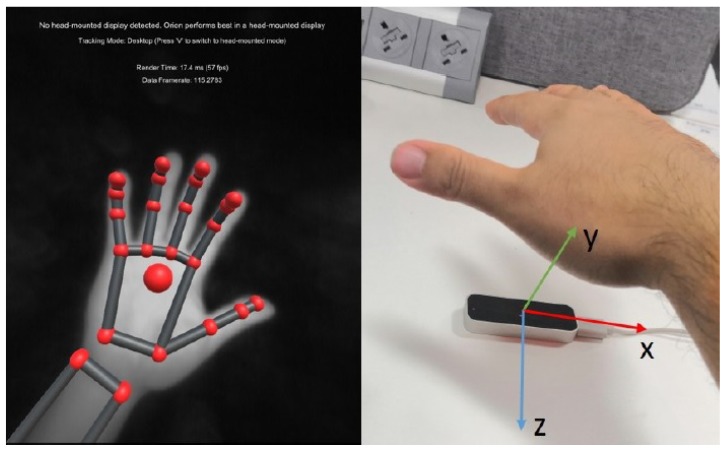
Leap Motion sensor with its Cartesian coordinate system and its visualizer function.

In this paper, we employ the Leap Motion somatosensor (shown in Figure 3) produced by Leap Motion Inc., an American somatosensory controller manufacturer in San Francisco, California, released in February 2013. It features a natural gesture function to control computer programs that replaces the need for traditional mouse and keyboard controls [25]. By acquiring user gestures in spatial motion data, we can naturally manipulate 3D objects using familiar gestures. The Leap Motion was a major breakthrough in accuracy, efficiency and portability with the accuracy of the Leap Motion being up to 200 times greater than other somatosensory products such as Microsoft’s Kinect [26]. It has been applied in many human-computer interaction roles, such as education, medicine, games, and Google Maps 3D, due to its intuitive nature and depth of control that can allow for the circumvention of the requirements for a mouse or touch screen.

Internally, a Leap Motion device consists of two narrow-band filters, two mid-low-pixel cameras, three infrared LEDs and a USB high-speed chip. The principle operation of Leap Motion is to capture a hand image illuminated by the infrared LED lamp through two cameras and establish a three-dimensional model of the hand by analysing the change of gesture in the stereo camera images. When the hand enters the field of view of the device, the stereo camera system begins capturing the target and calculates the parallax of the target in real time so that its spatial information is obtained [25]. The built-in communication chip in the body uses standard USB transmission technology to digitise the collected image information and transmit the converted data to the computer. The computer then restores the gesture changes through image recognition and calculation and resubmits hand motion to a PC application for use by the programmer.

**Figure 4:**
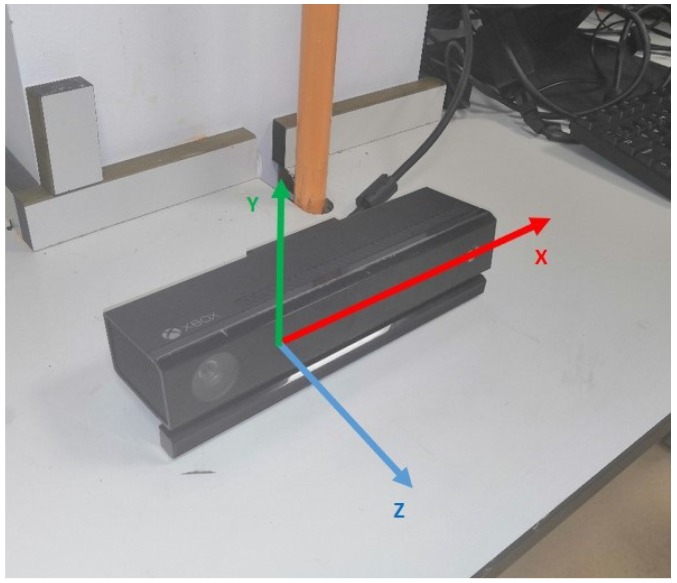
Illustration of the origin of the Kinect v2’s camera space. It is the same as its depth sensor origin.

The Kinect v2, released by Microsoft, is a camera sensor with RGB-D information and it can be used to capture IR images, colour and sound by developing new functions in the software development kit (SDK). The image and sound information is significantly better than those obtained using the first generation Kinect v1. Using the SDK, the obtained information can be transformed into camera space, which is in the real-world coordinates [27]. We can still employ the skeletal tracking function using the Kinect 2.0 SDK and this function has been widely used in our previous projects. Consequently, this technique is used to track the position data of a human operator, who needs to stand in front of the Kinect device. When a point in the Kinect colour image is selected by the operator, its depth information is acquired in the Kinect depth image. In the frame that is constructed, both the colour image, and depth image are put into the same frame and its origin is located at the centre of the depth camera. In this regard, the coordination system of the camera space follows a right-hand convention (see Figure 4).

## 3. Calibration

Before performing the tracking task, the transformation between the Baxter robot’s coordination and the LeapMotion’s is needed, according to our previous work [28], as shown in (1):

(1) $$X_{i}=TX_{i}^{{}^{\prime }}$$

where T denotes the transformation matrix. Hence, mathematically $X_{i}$ = ${\left[x_{i}y_{i}z_{i}1\right]}^{T}$ is the position data under the robot coordinates system, $X_{i}^{{}^{\prime }}$ = ${\left[x_{i}^{{}^{\prime }}y_{i}^{{}^{\prime }}z_{i}^{{}^{\prime }}1\right]}^{T}$ is the one under the LeapMotion system.

**Figure 5:**
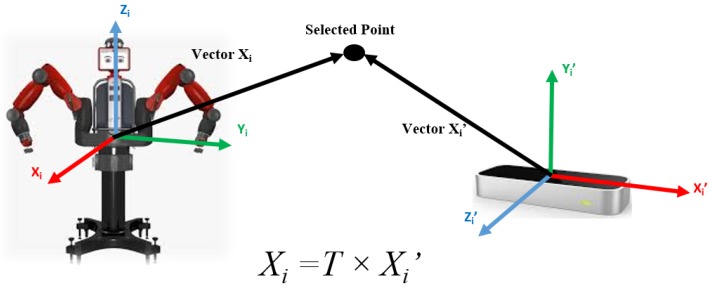
Illustration of the relationship between Baxter Robot and LeapMotion coordinate system.

The transformation matrix *T* can be calculated by selecting four points under both coordinates systems. Assuming the four points are—$p_{1}$, $p_{2}$, $p_{3}$ and $p_{4}$, lie in the robot coordinate system and the LeapMotion coordinate system, respectively. Figure 5, as an example, demonstrates one of those four points in the relationship between the Baxter robot and the LeapMotion’s coordinate system. Equation (2) illustrates the formula to obtain the transfer matrix *T* between the LeapMotion coordinates and the robot coordinates [29].

(2) $$T=\left[\begin{array}{cccc} x_{1} & x_{2} & x_{3} & x_{4}\\ y_{1} & y_{2} & y_{3} & y_{4}\\ z_{1} & z_{2} & z_{3} & z_{4}\\ 1 & 1 & 1 & 1 \end{array}\right]{\left[\begin{array}{cccc} x_{1}^{{}^{\prime }} & x_{2}^{{}^{\prime }} & x_{3}^{{}^{\prime }} & x_{4}^{{}^{\prime }}\\ y_{1}^{{}^{\prime }} & y_{2}^{{}^{\prime }} & y_{3}^{{}^{\prime }} & y_{4}^{{}^{\prime }}\\ z_{1}^{{}^{\prime }} & z_{2}^{{}^{\prime }} & z_{3}^{{}^{\prime }} & z_{4}^{{}^{\prime }}\\ 1 & 1 & 1 & 1 \end{array}\right]}^{-1}$$

In Reference [30], the authors presented a method that is able to obtain accurate calibration matrices for a robotic manipulator. There are still existing limitations for a dual arm robot, for example, the Baxter robot. It is difficult to achieve because the origin of the Baxter coordinate system is inside Baxter’s body, which is not visible and is not the same as the single arm based robotic manipulator whose coordinate system origin is located in the central point of the base. Thus, manual measurement using rulers to find the coordinate system is a commonly used method for the Baxter robot. However, it is hard to keep the ruler horizontal and vertical all the time. In this paper, to overcome the aforementioned issues, the kinematics of Baxter are employed. As a consequence of kinematics application, we can obtain the established reference coordinates and then order Baxter’s end-effector to move to these positions, from our previous work [28]. By doing this, the Baxter end-effector’s coordinates system can be obtained without measurement. After that, we use LeapMotion to define the Baxter end-effector’s coordinates in the LeapMotion’s coordinate system.

**Figure 6:**
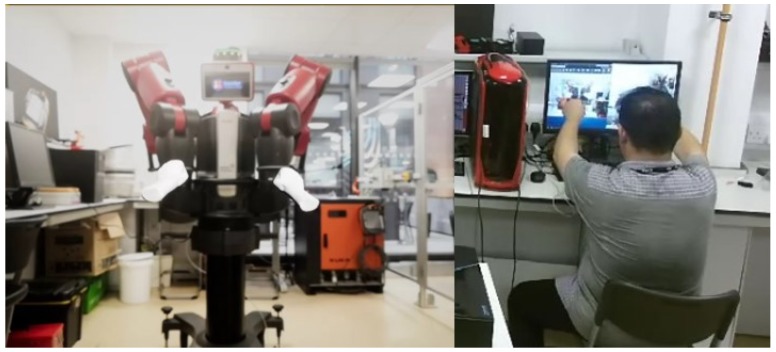
Illustration of the coordinates measurement process under the LeapMotion coordinate system.

Coordinates measurement process under the LeapMotion’s coordinate system was shown in Figure 6. To acquire the positions under the LeapMotion coordinate system, a person located in front of a table where a LeapMotion sensor is on it and put his hand over the LeapMotion to assure the virtual hand created by the Unreal Engine in the same position as that of the Baxter robot’s end-effector, which is for the LeapMotion sensor to collect the position data of the human operator’s hand palm in 3D space. The selected four positions under Baxter robot coordinate system are: $\left(x_{1},y_{1},z_{1}\right)$, $\left(x_{2},y_{2},z_{2}\right)$, $\left(x_{3},y_{3},z_{3}\right)$, $\left(x_{4},y_{4},z_{4}\right)$, which corresponding to the positions under the LeapMotion coordination: $\left(x_{1}^{{}^{\prime }},y_{1}^{{}^{\prime }},z_{1}^{{}^{\prime }}\right)$, $\left(x_{2}^{{}^{\prime }},y_{2}^{{}^{\prime }},z_{2}^{{}^{\prime }}\right)$, $\left(x_{3}^{{}^{\prime }},y_{3}^{{}^{\prime }},z_{3}^{{}^{\prime }}\right)$, $\left(x_{4}^{{}^{\prime }},y_{4}^{{}^{\prime }},z_{4}^{{}^{\prime }}\right)$, respectively. So far, the points’ coordinates in both Baxter’s and LeapMotion’s coordinate system in (2) are easily obtained.

However, when we apply kinematics, there are still stochastic errors existing. Owing to reducing these errors, the least squares method is introduced in Reference [29]. To find an overall solution for minimizing the sum of the square errors from the known data is the working principle of this algorithm. For the employment of the least squares method during the calibration process, it is ideal to transform (1) into the format in (3).

We define A=$\left[\begin{array}{cccc} x_{1}^{{}^{\prime }}I_{4} & y_{1}^{{}^{\prime }}I_{4} & z_{1}^{{}^{\prime }}I_{4} & I_{4}\\ x_{2}^{{}^{\prime }}I_{4} & y_{2}^{{}^{\prime }}I_{4} & z_{2}^{{}^{\prime }}I_{4} & I_{4}\\ x_{3}^{{}^{\prime }}I_{4} & y_{3}^{{}^{\prime }}I_{4} & z_{3}^{{}^{\prime }}I_{4} & I_{4}\\ x_{4}^{{}^{\prime }}I_{4} & y_{4}^{{}^{\prime }}I_{4} & z_{4}^{{}^{\prime }}I_{4} & I_{4} \end{array}\right]$, X=$\left[\begin{array}{c} T_{c1}\\ T_{c2}\\ T_{c3}\\ T_{c4} \end{array}\right]$, B=$\left[\begin{array}{c} x_{1}\\ y_{1}\\ z_{1}\\ 1\\ \ldots \\ x_{4}\\ y_{4}\\ z_{4}\\ 1 \end{array}\right]$, we can rewrite the equation below into [29]:

(3) AX=B

where A is a 16 × 16 dimension matrix, X denotes the 16 × 1 dimension transformation matrix *T*, *B* is a 16 × 1 dimension column vector, initially from [29]:

(4) $$\left[\begin{array}{cccc} x_{1}^{{}^{\prime }}I_{4} & y_{1}^{{}^{\prime }}I_{4} & z_{1}^{{}^{\prime }}I_{4} & I_{4}\\ x_{2}^{{}^{\prime }}I_{4} & y_{2}^{{}^{\prime }}I_{4} & z_{2}^{{}^{\prime }}I_{4} & I_{4}\\ x_{3}^{{}^{\prime }}I_{4} & y_{3}^{{}^{\prime }}I_{4} & z_{3}^{{}^{\prime }}I_{4} & I_{4}\\ x_{4}^{{}^{\prime }}I_{4} & y_{4}^{{}^{\prime }}I_{4} & z_{4}^{{}^{\prime }}I_{4} & I_{4} \end{array}\right]\left[\begin{array}{c} T_{c1}\\ T_{c2}\\ T_{c3}\\ T_{c4} \end{array}\right]=\left[\begin{array}{c} x_{1}\\ y_{1}\\ z_{1}\\ 1\\ \ldots \\ x_{4}\\ y_{4}\\ z_{4}\\ 1 \end{array}\right]$$

where $I_{4}\in R^{4\times 4}$ is the identity matrix, $T_{ci}$ is the column vector in transformation matrix.

Commonly, it is easy to find that the equation is difficult to obtain a solution; however, according to Reference [29], the least square solution of this equation can be calculated. Subsequently, the solution of Equation (4) can be obtained by substituting $\left(x_{i},y_{i},z_{i}\right)$ and $\left(x_{i}^{{}^{\prime }},y_{i}^{{}^{\prime }},z_{i}^{{}^{\prime }}\right)$ (i = 1,2,3,4) in, which is employed to collect the operator’s hand palm centroid position. These position signals are sent to fuse with the Kinect V2 camera by KF.

## 4. Measurement of Montion Velocity

### 4.1. Depth Information Acquisition

The depth data that the Kinect V2 camera can acquire has the shortest and largest distances, but the accuracy of the depth data after 4 m from the camera will decrease. In order to obtain accurate data, we choose to detect near the shortest detection range of the Kinect V2 camera. The method used in this paper is to first calculate the minimum value of all the depth data obtained and then calculate other depth data based on this. The advantage of this is that the range of data acquired can change dynamically when the distance from the camera changes.

### 4.2. Pixel Matrix Generation

This step requires processing the depth data, which is the data that is used for tracking. Kinect V2 offers three resolutions which are 80×60, 320×240 and 640×480. In order to reduce the amount of calculation, here 320×240 resolution is selected. This resolution is sufficient for distinguishing between human hands and also improves code execution efficiency. Because algorithms consider the relationship of adjacent points, once the depth data is acquired, a matrix of the corresponding size should be generated. The boundary value of the matrix is set as “False”, which eliminates the problem of checking whether the access is out of bounds every single time.

### 4.3. Hand Palm Tracking

In this paper, the skeleton of the human body is tracked by Kinect V2 camera sensor to obtain the spatial coordinates of the joint points of the human palm center in the camera space using Kinect SDK 2.0. Assuming *x*, *y*, *z* coordinates are the operator’s hand palm centroid position vectors over n frames, the instantaneous motion velocity of the human palm center can be calculated as [31]:

(5) $$\begin{array}{cc} u & =\frac{d_{x,y,z}}{dt}{|}_{t=\frac{n}{fs}}\\ \; & =fs\times \sqrt{{\left[x\left(n\right)-x\left(n-1\right)\right]}^{2}+{\left[y\left(n\right)-y\left(n-1\right)\right]}^{2}+{\left[z\left(n\right)-z\left(n-1\right)\right]}^{2}} \end{array}$$

where fs denotes the sampling frequency, *n* is the number of frames, *u* is computed as a result of the *x*, *y*, and *z* positions on all frames representing motion. The velocity component vectors of *u* in 3 directions are defined $u_{x}$, $u_{y}$, $u_{z}$, respectively, which are used to fuse with the positions signals outputted by LeapMotion.

## 5. Kalman Filtering Based Sensor Fusion

The core idea of the KF algorithm is to calculate the current optimal amount based on the current “measured value”, the “predicted value” and the "error" of the previous moment and then predict the value of the next moment. The more prominent point is that the error is included in the calculation and it is divided into two types—prediction error and measurement error, which is commonly called noise. If there is a linear dynamic model, the noises can be represented using white noise models that obey the Gaussian distribution. Another notable feature is that the error exists independently and is always unaffected by the measured data. In this regard, KF is able to fuse data with a unique statistically optimal estimate. The recursive KF reduces the computational burden and it is divided into two types—continuous-time KF and discrete KF.

The temporal mathematical model of the continuous-time KF is produced below [32]:

(6) $$\begin{array}{cc} \; & \dot{X_{sv}}\left(t\right)=A\left(t\right)X_{sv}\left(t\right)+B\left(t\right)u_{sv}\left(t\right)+G\left(t\right)W\left(t\right)\\ \; & Z_{mv}\left(t\right)=H\left(t\right)X_{sv}\left(t\right)+V\left(t\right) \end{array}$$

where $X_{sv}\left(t\right)$=${\left[x\left(t\right)\;y\left(t\right)\;z\left(t\right)\right]}^{\prime }$, $u_{sv}\left(t\right)$=${\left[u_{x}\left(t\right)\;u_{y}\left(t\right)\;u_{z}\left(t\right)\right]}^{\prime }$ are the *n*-dimensional state vectors at time *t*; $Z_{mv}\left(t\right)$ is the measurement vector with *m*-dimension at time t; *A* (n×n), *G* and *B* (n×r both) denote system matrices; *H* is the measurement matrix with m×n-dimensional; *W* represents the *n*-dimension continuous system’s white noise vector; *V* is an *r*-dimension vector denoting the potential white noise among the measured data.

**Figure 7:**
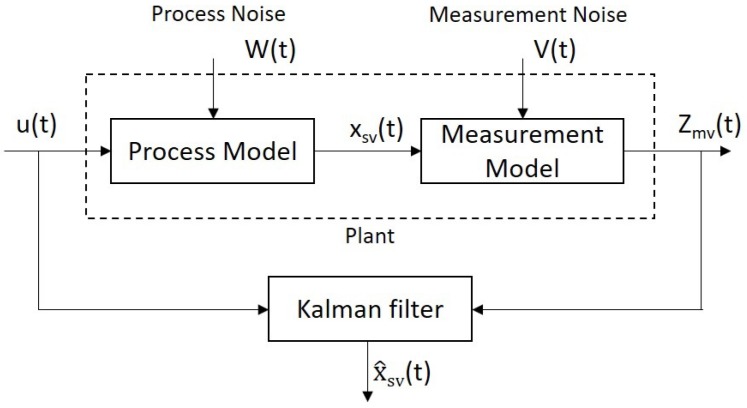
Illustration of the continuous-time KF diagram.

The continuous-time KF status equation, based on Reference [33] is stated as follows,

(7) $$\begin{array}{cc} \; & \dot{\hat{X_{sv}}}\left(t\right)=A\left(t\right)\hat{X_{sv}}\left(t\right)+B\left(t\right)u_{sv}\left(t\right)+K\left(t\right)\left[Z_{mv}\left(t\right)-H\left(t\right)\hat{X_{sv}}\right]\\ \; & K\left(t\right)=P\left(t\right)H^{T}\left(T\right)\Gamma^{-1}\left(t\right)\\ \; & \dot{P}\left(t\right)=P\left(t\right)H^{T}\left(t\right)+A\left(t\right)P\left(t\right)-P\left(t\right)H^{t}\left(t\right)\Gamma^{-1}H\left(t\right)P\left(t\right)\\ \; & +G\left(t\right)s\left(t\right)G^{T}\left(t\right) \end{array}$$

where *K* is the gain matrix of KF, *P* is the estimated covariance matrix and $\hat{x}$ is the estimated value of *x*. We are able to obtain the continuous-time KF, once we have the measured values of the continuous-time series. This solution is required to estimate the system state variable, which solves the differential equation of the matrix. Moreover, the continuous-time KF does not require complex recursive calculations. The working principle of KF is illustrated in Figure 7.

In order to employ the KF algorithm, there are two standard requirements that need to be satisfied: (1) the system model is dynamically time-varying excited by white noise; (2) there are white noise components existing in the measurement signal.

In this paper, the pre-collected signals from the LeapMotion sensor and Kinect V2 camera fulfilled the above stated requirements, hence we incorporate signals from multiple sensors via the continuous-time KF algorithm.

Assuming $X_{sv}\left(0\right)$, *W* and *V* are not related to each other, in References [32,33], the expected values of the continuous-time KF satisfy the following statistical characteristics,

(8) $$\begin{array}{cc} \; & E\left\{X_{sv}\left(0\right)\right\}=e\left(0\right);\\ \; & E\left\{W(t)\right\}=E\left\{V(t)\right\}=0;\\ \; & E\left[X_{sv}\left(0\right)-e\left(0\right)\right]{\left[X_{sv}\left(0\right)-e\left(0\right)\right]}^{T}=P\left(0\right);\\ \; & E\left\{X_{sv}\left(0\right)W^{T}\left(t\right)\right\}=E\left\{X_{sv}\left(0\right)V^{T}\left(t\right)\right\}\\ \; & =E\left\{W\left(t\right)V^{T}\left(t\right)\right\}=0;\\ \; & E\left\{W\left(t\right)W^{T}\left(\tau_{KF}\right)\right\}=s\left(t\right)\delta \left(t-\tau_{KF}\right);\\ \; & E\left\{V\left(t\right)V^{T}\left(\tau_{KF}\right)\right\}=\Gamma \left(t\right)\delta \left(t-\tau_{KF}\right); \end{array}$$

where Γ and *s* represent the continuous system’s measurement noise variance intensity and its noise variance intensity matrix, receptively; P(0) denotes the initial covariance matrix of the state vector $X_{sv}$; e(0) is its the initial mean value; $\delta (t-\tau_{KF})$ is the Dirac δ function.

We assume that every single joint of the human arm is taken into account separately and not depending on each other, which shows that all the KF parameters are in first order. According to our previous work [34], this is the main reason behind the de-coupling of the different position coordinates within the KF, thus we have *A*=*B*=*G*=*H*=1. In addition, since the measurement errors are not correlated in the *x* direction, the *y* direction and the *z* direction, the filtering can be performed separately in three directions. The KF equations are simplified as below,

(9) $$\begin{array}{cc} \; & \dot{x}\left(t\right)=x\left(t\right)+u_{x}\left(t\right)+W\left(t\right)\\ \; & \dot{y}\left(t\right)=y\left(t\right)+u_{y}\left(t\right)+W\left(t\right)\\ \; & \dot{z}\left(t\right)=z\left(t\right)+u_{z}\left(t\right)+W\left(t\right)\\ \; & Z_{mv,x}\left(t\right)=x\left(t\right)+V\left(t\right)\\ \; & Z_{mv,y}\left(t\right)=y\left(t\right)+V\left(t\right)\\ \; & Z_{mv,z}\left(t\right)=z\left(t\right)+V\left(t\right) \end{array}$$

where in this special case, $Z_{mv,x}$, $Z_{mv,y}$, $Z_{mv,z}$ are the arm end-effector’s position along *x*, *y* and *z* directions, respectively, collected by the LeapMotion sensor. And $u_{x}$, $u_{y}$ and $u_{z}$ are the 3 directions related motion velocities of hand palm, collected from Kinect V2 camera.

(10) $$\begin{array}{cc} \; & \dot{\hat{x}}\left(t\right)=\hat{x}\left(t\right)+u_{x}\left(t\right)+K\left(t\right)\left(Z_{mv,x}\left(t\right)-\hat{x}\left(t\right)\right)\\ \; & \dot{\hat{y}}\left(t\right)=\hat{y}\left(t\right)+u_{y}\left(t\right)+K\left(t\right)\left(Z_{mv,y}\left(t\right)-\hat{y}\left(t\right)\right)\\ \; & \dot{\hat{z}}\left(t\right)=\hat{z}\left(t\right)+u_{z}\left(t\right)+K\left(t\right)\left(Z_{mv,z}\left(t\right)-\hat{z}\left(t\right)\right)\\ \; & K\left(t\right)=P\left(t\right)\Gamma^{-1}\left(t\right)\\ \; & \dot{P}\left(t\right)=2P\left(t\right)-P\left(t\right)\Gamma^{-1}\left(t\right)P\left(t\right)+s\left(t\right) \end{array}$$

where *K* is the filter gain matrix along one of those 3 directions, *P* is the estimated covariance matrix along one of those 3 directions, $\hat{x}$, $\hat{y}$ and $\hat{z}$ are the required (satisfied) data obtained from KF based sensor fusion for all of the *x*, *y* and *z* directions, which are also the statistics that needs to be sent to the robot for the hand motion following via UDP.

## 6. Experimental Study & Aanlysis

### 6.1. Experimental Setup

**Figure 8:**
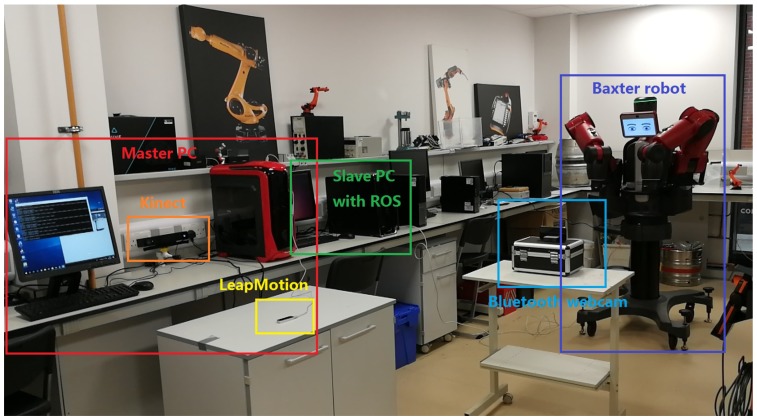
The experimental human robot interaction interface.

Several experimental studies have been conducted to validate the tracking performance of the our proposed method. The experimental platform includes: PC operation system windows 10, Kinect SDK 2.0 for Windows and Visual studio 2017. The experiment environment is an indoor and adequate illumination environment. One person seats aside by the LeapMotion sensor and put his hand over it, which is on a table. A Kinect V2 camera is facing the human operator to measure the velocity of the hand motion. A bluetooth webcam is put opposite the Baxter robot centrally to match the workspace between the Baxter end-effector and the pair of virtual hands. After all the above steps had been completely set up, as shown in Figure 8, the LeapMotion controller and the AR environment made by Unreal Engine then can be run, a view of Baxter and a pair of virtual hands tracked by LeapMotion sensor can be seen as shown in Figure 6. When the operator grasps his hands, the arms of the Baxter start to mirror the hand motion of the operator.

First of all, a blind test was taken to assess the easiness of the developed teleoperation system, where ten participants performed a set of different hand gestures five times each. There are only two gestures involved in this paper—the fist and the outstretched palm. When the operators made a fist, the robot started to be controlled and when they outstretched their hands, the robot stopped moving. Operators need to make fists during control of the Baxter robot. Thus, there are only two gestures that need to be detected.

In the second experiment, the operator smoothly moved his hands over the LeapMotion while making fists. The hand position data were sent to teleoprate the Baxter via UDP directly. At the same time, those data and their corresponding motion velocity of both hands’ palms were outputted by the LeapMotion and Kinect V2, respectively, to local files for recording and experimental result analysis purposes. For the third experiment, the recorded data from the second experiment were loaded to be fused by the KF algorithm, which was programmed by the Simulink function of MATLAB. The fused/optimal data were then sent to teleoperate the Baxter robot in real-time and outputted to local for recording as well.

The LeapMotion sensor had its own limitation. It is based on image recognition tracking and there are errors in the tracking process as the LeapMotion sensor has a limited light cover range. To keep the tracking accurate, the operator needs to keep his hand always in a small movable space. More significantly, there are still sensor noises; thus, the KF is employed to fuse the position data in 3D space collected from the LeapMotion sensor with the corresponding motion velocity measured by the Kinect camera sensor to reduce the sensor noises to the lowest level.

### 6.2. Experimental Result & Analysis

**Figure 9:**
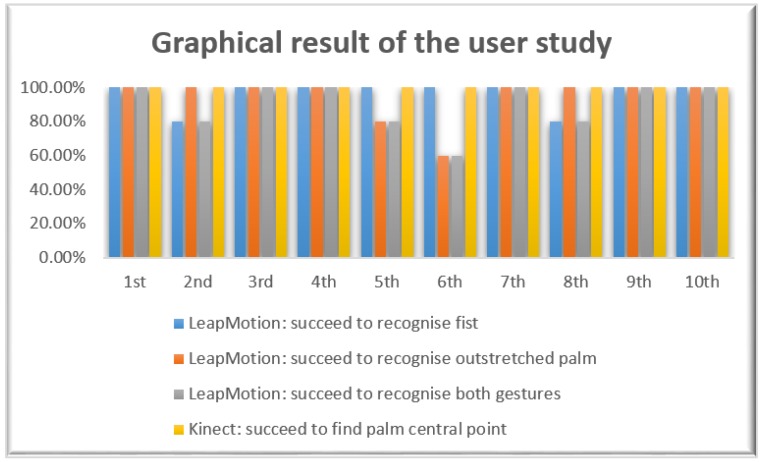
The bar chart graph of the blinded test result.

Ten participants took turns to stand in front of the Kinect V2 camera and performed different gestures casually by putting their hands over the LeapMotion sensor. In order for the proposed tracking system to work well, the LeapMotion sensor needs to recognise the fist and outstretched palm gestures and the Kinect V2 needs to recognise the hand palm central point first. Figure 9 illustrates the result of the user study.

We can see from the bar chart, except for the second, fifth, sixth and eighth participants, all the other participants’ blind tests were fully passed. For the second and eighth participants, they did not perform the fist gesture during the test; the fifth participant did not perform the outstretched palm gesture. The sixth participant performed the casual gestures five times including those 2 desired gestures, however, our proposed tracking system failed to recognise the outstretched palm gesture twice. This may have been caused by the hand fluctuation or the over-fast switching between different gestures. It can be summarized that our proposed teleoperation system has high detection accuracy.

**Figure 10:**
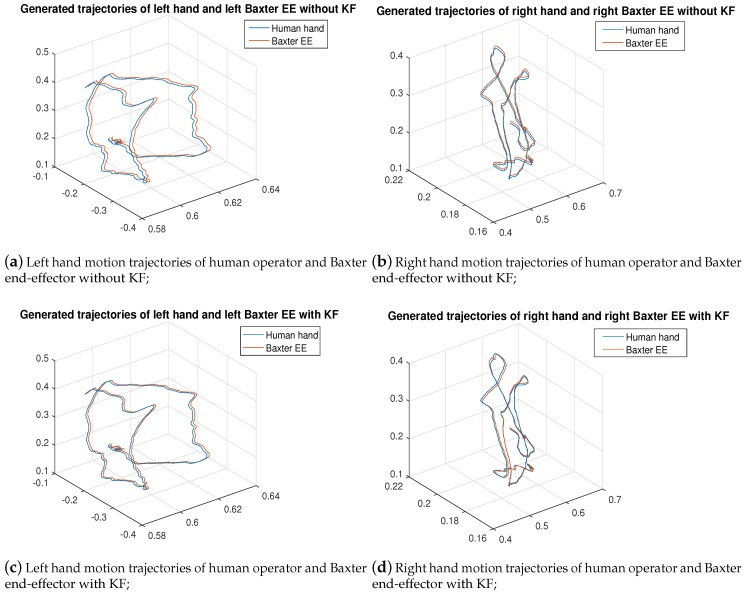
Graphical result of both hands between the human and robot with & without KF sensor fusion (in m).

**Figure 11:**
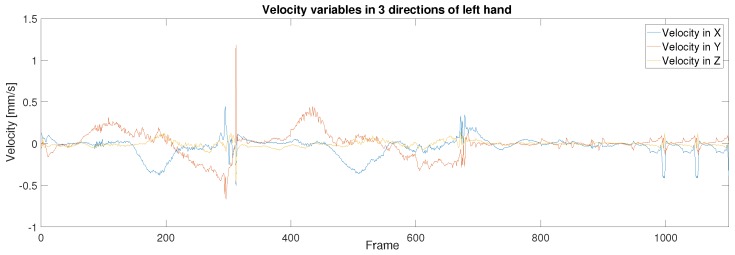
Motion velocity of the left hand of the human operator in 3 directions.

**Figure 12:**
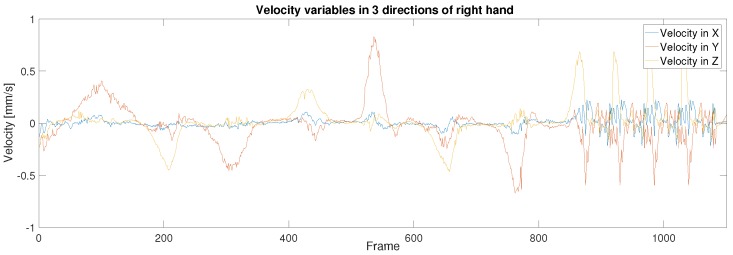
Motion velocity of the right hand of the human operator in 3 directions.

In order to start the control process, the operator was required to use their hands to seek and grasp the Baxter end-effector in the AR environment instead of in actual reality, where the points of virtual palms made by the Unreal Engine are difficult to completely overlap with the central point of Baxter’s end-effector in the display window. Thus, there is a displacement between the trajectories. Figure 10 illustrates the experimental results of the trajectories for both the human hand palm and the Baxter’s end-effector after the application of the KF-based sensor fusion. The LeapMotion sensor provides the real position trajectory of the human hands. Figure 10a,b represent the original position data of the human operator’s hands without the KF sensor fusion directly collected from the LeapMotion sensor; Figure 10c,d represent the optimal position data after the KF sensor fusion. From the images above, we can see that the trajectories for both hands were better fitted by applying the KF based sensor fusion. Figure 11 and Figure 12 demonstrate the real-time instantaneous motion velocities of the human hands in 3 directions. The Kinect camera provides the motion velocities along *x*, *y*, *z* directions accordingly before they are fused together via KF.

The average ratio of position data difference in Cartesian space between the human hand palm and the Baxter’s end-effector and the original raw position data obtained from LeapMotion was selected as the performance index, which was defined as $r_{e}$ as formulated in Equation (11). Table 1 represents how the performance index changed with and without the KF based sensor fusion for all 3 directions.

(11) $$r_{e}=\frac{\left|Human_{x,y,z}-Baxter_{x,y,z}\right|}{Human_{x,y,z}}$$

where $Human_{x,y,z}$ and $Baxter_{x,y,z}$ are the experimental position data in Cartesian space of the human operator hand palm and the end-effector of the Baxter robot, respectively. From Table 1 we can see that the Baxter robot with KF sensor fusion can follow the human operator’s motion with the promotion ratio at about 33% in all 3 directions, compared to the situation without KF sensor fusion technology. We can draw a conclusion that the proposed tracking system has better performance after employing the KF based sensor fusion.

### 6.3. Remark

The usage of a LeapMotion and a Kinect after KF based sensor fusion to interact with a Baxter robot has been developed and verified. From the first experiment, the conclusion can be drawn that our proposed tracking system has great recognition performance for hand gestures from different users. For the second and third experiments, the results shown in Table 1 demonstrate a series of ratios, which are the promotion percentages in 3 axis directions between those returned by the Baxter robot and those directly collected from LeapMotion because the values filtered by KF are optimally estimated and the noise during the teleoperation process is suppressed. The performance index shown in Table 1 is averagely promoted at about 33% after employing the KF based sensor fusion. Thus, it can be summarised that our proposed teleoperation strategy has better tracking performance after the application of the KF based sensor fusion. How to improve the robustness of the proposed control system against control parameters, and reduce the overall sensitivity of the method relative to parameter perturbations will be systematically investigated in our future work.

## 7. Conclusions

In this paper, an AR based human-robot interaction technology has been developed. Unreal Engine is used to create a pair of virtual hands tracked by the LeapMotion sensor. The LeapMotion is also used to collect the central point position of the human hand palm and map with the end-effector of the Baxter robot for the hand motion following the task. In addition, a KF based sensor fusion method is used to improve the tracking performance. This involved using the LeapMotion sensor to capture the motion of an operator’s hand after the calibration process. Moreover, the velocities in 3 axis directions of the human operator’s hand motion is measured by the Kinect V2 camera. The fused data are thereafter sent to the Baxter robot for teleoperation. Experimental studies have been conducted to test the validation of our proposed method. The experimental results show that the performance index evidently changes after the application of KF based sensor fusion, and the employment of the AR environment greatly enhanced the user experiences during the teleoperation process.

## Figures and Tables

**Table 1:** Performance index table in different directions with & without KF sensor fusion.

Performance Index Values	Without KF		With KF		Promotion Ratio	
	Left	Right	Left	Right	Left	Right
*x* direction	5.297%	2.652%	3.587%	1.763%	32.272%	33.522%
*y* direction	5.438%	2.786%	3.609%	1.815%	33.628%	34.852%
*z* direction	4.973%	2.541%	3.304%	1.655%	33.573%	34.868%
